# How hot is too hot for people? A review of empirical models of perceptual, physiological and functional limits of human heat tolerance

**DOI:** 10.1113/EP092242

**Published:** 2025-08-25

**Authors:** Davide Filingeri, Nuno Koch Esteves

**Affiliations:** ^1^ ThermosenseLab, Skin Sensing Research Group, School of Health Sciences University of Southampton Southampton UK; ^2^ Tyndall Centre for Climate Change Research University of Southampton Southampton UK; ^3^ Division of Sport, Health and Exercise Sciences, Department of Life Sciences Brunel University of London Uxbridge UK

**Keywords:** body temperature, global warming, humans, policy, thermotolerance

## Abstract

How hot is too hot for people? This is a question that human thermal physiologists are asked often by a variety of knowledge users across the public and private sectors, who have grown aware of the negative impact of global warming on people's health and quality of life. The aim of this paper is to provide a narrative review of models that quantify the limits of human heat tolerance across perceptual, physiological and functional domains. Several models exist that have identified critical environmental limits for heat tolerance across the perceptual, physiological and functional domains. However, no model is currently available that has evaluated all domains of heat tolerance concurrently and in the same participant cohort. Hence, by combining evidence from these models, here we propose a new holistic framework of heat tolerance that can help more comprehensively characterise the full spectrum of possible human responses to heat stress under free‐living conditions. This framework highlights that human heat tolerance varies largely across the perceptual, physiological and functional domains, and that it is conceptually organised in line with the human body's ability to regulate body temperature via behavioural and autonomic responses. While our new framework presents limitations in its generalisability beyond healthy young adult cohorts, we hope that it will inspire the design of new holistic research on human heat tolerance in a broader range of participant cohorts, to better inform person‐centred heat resilience policies and interventions that protect human health and life quality under a warming climate.

## Abstract (highlights)

- **What is the topic of this review?**
  How hot is too hot for people?
- **What advances does it highlight?**
  Using evidence from models that have identified critical environmental limits for heat tolerance across the perceptual, physiological and functional domains, we propose a new holistic framework of heat tolerance that can help more comprehensively characterise the full spectrum of possible human responses to heat stress under free‐living conditions.

## 1 INTRODUCTION

In our capacity as human thermal physiologists, we are asked very often ‘how hot is too hot for people?’ by a variety of knowledge users across the public and private sectors. This question has become increasingly more frequent since people and organisations worldwide have grown aware of the negative impact of global warming on people's health and quality of life (van Daalen et al., 2024).

Sometimes the question ‘how hot is too hot?’ is posed by architects from local government authorities, who need to develop care homes that are ‘heat‐proof’ and safer for a variety of vulnerable residents (e.g., young, old, and/or with mental or physical disabilities) (UK Health Security Agency, 2025), during the more frequent heatwaves that we have experienced in recent years (Gallo et al., 2024). Sometimes the question is posed by textile engineers from global sportwear manufacturers, whose goal is to design clothing that helps people of all ages and abilities to stay more comfortable and active in increasingly warmer environments (Di Domenico et al., 2022). Other times, the question is posed by project managers from local hospitals, who oversee the design of surgical theatres and wards that must meet the complex care needs of patients and the healthcare workforce, as well as the net‐zero targets for energy efficiency in the provision of indoor thermal comfort (Brooks et al., 2023).

A seemingly simple question, such as ‘how hot is too hot?’ can rarely be met with a simple answer. One may argue that the first, necessary step to provide a conclusive answer on people's tolerance of heat is to clearly define those ecologically valid, free‐living conditions and scenarios, within which the thermal comfort, thermal health and/or thermal safety of such people is to be achieved. Yet, framing the context within which heat tolerance is being evaluated can only partly facilitate the provision of an answer to the question ‘how hot is too hot?’. An essential prerequisite for our ability to address this question conclusively is a comprehensive understanding of the concept of *human heat tolerance* and its underlying mechanisms.

**FIGURE 1:**
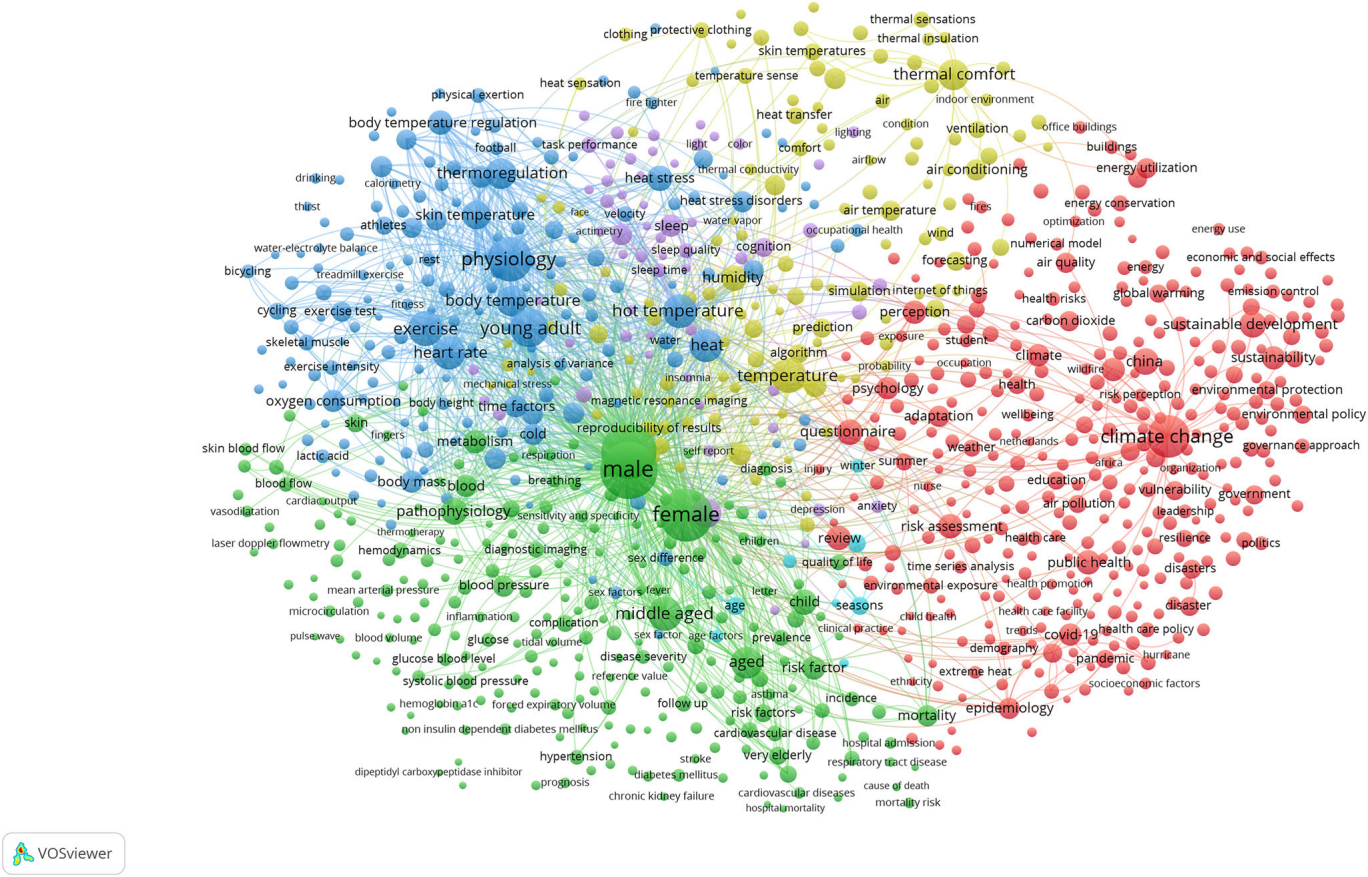
Word cloud illustrating the most frequently mentioned terms related to heat tolerance. Larger words represent a higher occurrence of themes in the literature. Word cloud conducted on 9 December 2024. The interactive word cloud can be found here: https://tinyurl.com/26k46xnh.

The last century has seen a significant increase in our understanding of the basic mechanisms of human physiological responses to heat stress (Cramer et al., 2022), partly due to the growing interest in the impact of climate‐change‐induced global warming on human health. A brief search of relevant literature over the last decade (i.e., since the Paris COP21 agreement in 2015), using keywords associated with human heat tolerance (see Appendix), and the associated word cloud visualisation of relevant keyword clusters (Figure 1), shows how knowledge on the nexus between heat stress and health is largely rooted in research addressing the physiology of body temperature regulation (i.e., Figure 1, blue cluster – top left quadrant) and (to a lesser extent) thermal comfort (i.e., Figure 1, yellow cluster top centre quadrant). However, the physiology of body temperature regulation during heat exposure has been mostly studied in isolation from people's perceptual and functional capacity to respond and adapt to heat stress, both of which are cornerstones of behavioural thermoregulation (Vargas et al., 2023). This is surprising, given that under free‐living conditions, behavioural thermoregulation, defined as ‘any coordinated movement of an organism ultimately tending to establish a thermal environment that represents a preferred condition for heat exchange (heat gain, heat loss, or heat balance) of the organism with its environment’ (The Commission for Thermal Physiology of the International Union of Physiological Sciences, 2001), represents humans’ first line of defence from the heat (Schlader & Vargas, 2019).

Increasing our holistic understanding of human heat tolerance, by considering the complex interplay amongst perceptual, physiological and functional responses to heat, has been recently highlighted as an increasingly urgent global priority (van Daalen et al., 2024). This knowledge could support the development of evidence‐based, person‐centred solutions and technologies that better protect the most vulnerable and under‐represented people in our society from heat stress (e.g., children, the elderly and those with long term physical and mental health conditions) (van Daalen et al., 2024). This is a particularly important consideration, given that under‐represented groups experience a disproportionality greater and more negative impact of global warming on their health and life quality (Gulcebi et al., 2025; Stein & Stein, 2022).

The aim of this paper was to provide a narrative review of empirical models and approaches that quantify the limits of human heat tolerance across perceptual, physiological and functional domains. We will use this evidence to propose a holistic framework of human heat tolerance that could be used to more comprehensively characterise the full spectrum of possible human responses to heat stress under free‐living conditions. We hope that this framework will inspire the design of new holistic research on human heat tolerance, which will ultimately inform heat resilience policies and interventions to protect human health and life quality under a warming climate.

## 2 THE IUPS DEFINITION OF (HUMAN) HEAT TOLERANCE

The availability of clear definitions of fundamental concepts is essential for any scientific field to further the understanding of a topic of interest. Thermal physiology is no exception to this rule, and colleagues in our field often refer to the latest version of the glossary of terms for thermal physiology, which is compiled by the Commission for Thermal Physiology of the International Union of Physiological Sciences (IUPS), to guide trainees in their understanding of basic concepts of body temperature regulation (The Commission for Thermal Physiology of the International Union of Physiological Sciences, 2001).

In its third edition of the glossary of terms for thermal physiology, the Commission broadly defines heat tolerance as the ‘ability to tolerate high ambient temperatures’. In the case of tachymetabolic homeothermic species such as humans, the IUPS definition specifies that ‘[these species are characterised as heat tolerant] if they remain comfortable or are able to balance heat production and heat loss at particularly high ambient temperature. Homeothermic species are also characterized as heat‐tolerant if they are capable of maintaining normal functions, or to survive, at body temperatures exceeding their normal range’.

As currently reported, the IUPS definition of heat tolerance can be interpreted to indicate that a species is considered heat tolerant if any of the following three (interrelated) criteria are met:

1. The maintenance of comfort at particularly high ambient temperature.
2. The maintenance of heat balance at particularly high ambient temperatures.
3. The maintenance of normal functions or survival at body temperatures exceeding their normal range.

Aspects of this definition are somewhat open to interpretation. For example, ‘normal functions’ may relate to the full operation of vital physiological processes as well as to normal tasks of daily living, including work. The Commission also clearly notes the ambiguity associated with the use of the term heat tolerance as a synonym of ‘heat endurance’, which indicates the ability to withstand a sustained stress (e.g., exercise in the heat). Furthermore, this definition does not directly address the complex interplay between ambient temperature and humidity in modifying the ability to maintain heat balance (i.e., criterion 2); ambient humidity is well known to be an important factor for species such as humans, who rely on the evaporation of sweat as their most effective avenue of heat loss to the environment (Baldwin et al., 2023).

Despite the limitation highlighted above, the IUPS definition and related criteria effectively identify three general forms of heat tolerance for tachymetabolic homeothermic species such as humans:

1. Perceptual heat tolerance (arising from the ‘comfort’ criterion 1).
2. Physiological heat tolerance (arising from the ‘heat balance’ criterion 2).
3. Functional heat tolerance (arising from the ‘function/survival’ criterion 3).

The availability of an agreed definition of heat tolerance, which encompasses the full spectrum of possible responses to heat stress, that is, perceptual, physiological and functional, offers an important conceptual framework to operationalise the limits of human heat tolerance. For example, the definitions of the first two forms of heat tolerance (i.e., perceptual and physiological) are associated with ambient temperature; one could therefore use either of those two definitions to identify ambient temperatures beyond which comfort and/or heat balance can no longer be maintained and describe them as operational limits to human (perceptual and/or physiological) heat tolerance. Furthermore, the third definition of heat tolerance (i.e., functional) is associated with body temperature; one could therefore use this definition to identify body temperatures beyond which normal function (however defined) or survival can no longer be maintained and describe them as operational limits to human (functional) heat tolerance.

The three IUPS criteria of heat tolerance are intrinsically connected, meaning that under real‐life scenarios of heat exposure, an individual is likely to concurrently experience varying degrees of change in their comfort, heat balance and function. This observation further highlights the complexity of addressing the question ‘how hot is too hot for people?’ with a single answer that can meaningfully accommodate the perceptual, physiological and functional domains of human heat tolerance. It is also important to note that the availability of a general definition of heat tolerance does not necessarily translate into its consistent use and implementation within empirical or modelling studies that may have informed our current understanding of human responses to heat.

The following sections will present relevant empirical models that have helped quantify the perceptual, physiological and functional limits of human heat tolerance, by reviewing their underlying approaches through the lens of the cited IUPS definition of human heat tolerance.

## 3 EMPIRICAL MODELS OF PERCEPTUAL HEAT TOLERANCE

The first IUPS criterion of human heat tolerance concerns the maintenance of comfort at particularly high ambient temperatures. As noted earlier, one could use this definition to identify ambient temperatures beyond which comfort can no longer be maintained and describe them as operational limits to human perceptual heat tolerance. This approach has long been adopted in the study of indoor thermal comfort within the built environment, and the interested reader is referred to a recent excellent review by de Dear et al. (2020) for a broader context.

The primary purpose of assessing the limits of human perceptual heat tolerance within the built environment is to inform the design of indoor environments (e.g., offices) that maintain occupants’ thermal comfort (van Hoof, 2008). As a result, the engineering and architecture disciplines have contributed the most to the development of empirical evidence in support of our current understanding of thermal comfort and associated models (de Dear et al., 2020). For example, the first scientific approach to the study of thermal comfort was conducted by Houghton et al. within the American Society of Heating and Ventilating Engineers (ASHVE) research laboratory at Pittsburgh, USA. For a (quasi‐) historical perspective on this matter, the reader is referred to the classic paper of Gagge et al. (1967).

Given the influential role of the engineering and architecture disciplines in our current understanding of thermal comfort, it is not entirely surprising that the most widely accepted and used definition of thermal comfort stems from three international standards for occupant comfort within the built environment, that is, ASHRAE 55–2023, ISO 7730:2005 and EN 16798–1:2022 (ASHRAE, 2025; Deutsches Institut für Normung, 2025; ISO, 2005). Within those standards, thermal comfort is defined as ‘the condition of mind that expresses subjective satisfaction with the thermal environment’. This definition is widely accepted and used also within the thermal ergonomics and physiology fields (Parsons, 2014), despite the IUPS glossary of terms reporting a different definition of thermal comfort (i.e., ‘subjective indifference to the thermal environment’).

The familiarity and widespread use of this engineering‐derived definition of comfort within the thermal ergonomics and physiology fields is likely the result of the influential work of A. Pharo Gagge, a world‐renowned biophysicist and pioneer investigator of the interaction of varied environments with human body temperature, and who championed interdisciplinary collaboration in the thermal biophysics and bioengineering areas (Von Gierke, 2025). This is perhaps most evident in Gagge's influential role and work across both the American Society of Heating, Refrigerating and Air‐Conditioning Engineers (ASHRAE) (see, e.g., Gagge et al., 1971) and the American Physiological Society (APS) (see, e.g., Gagge & Gonzalez, 1996).

This brief contextual overview on the origins of the definition of thermal comfort across building engineering and thermal physiology is important, as it provides the rationale for the widespread use of the cited international standards (i.e., ASHRAE 55–2023, ISO 7730:2005 and EN 16798–1:2022) to model occupant comfort within the built environment.

Those international standards are based on two influential, empirical models of human thermal comfort, namely (1) the Predicted Mean Vote (PMV) model by Fanger (1972), and the Adaptive Comfort model by de Dear and Brager (1998). Aside from their relevance to the engineering fields, these models are important as they identify ambient temperatures (in combination with relative humidity) beyond which comfort can no longer be maintained, which can be used to describe the operational limits of human perceptual heat tolerance. It is important to note that, while the IUPS criterion associated with perceptual heat tolerance does not directly address the role of ambient humidity, the latter environmental parameter has been long known to play a significant role in warm discomfort (Gagge, 1937; Gagge et al., 1967), and it is therefore incorporated in comfort models such as the PMV.

Both the PMV and Adaptive Comfort models have been extensively reviewed by others, including the excellent work of van Hoof (2008) and de Dear et al. (2020), and it is beyond the scope of the current review to comprehensively discuss the use and limitations of each model. It is, however, worth highlighting the general principles underlying each approach.

As eloquently summarised by de Dear et al. (2020), Fanger's PMS model is [cit.] ‘a deterministic model …, in which the relationship between building occupant and their indoor climate is construed as a simple balance between the body's metabolic heat production on the one hand, and the transfer of that heat to the indoor environment through convective, conductive, radiative and latent heat fluxes, on the other’. Specifically, the main principle of Fanger's PMV model is that the human body should meet a number of conditions in order to perceive (steady‐state) thermal comfort, that is, (1) the body is in heat balance, (2) mean skin temperature and sweat rate are within certain limits and (3) no local discomfort exists (Fanger, 1972). It is important to note that the PMV model is a ‘population‐based’ model which ultimately predicts the mean thermal sensation vote for a large group of persons, and it indicates the deviation from presumed ‘optimal’ thermal comfort, that is, thermoneutrality; to do so, the model considers the absolute and relative impact of six factors, namely, air temperature, mean radiant temperature, air velocity, relative humidity, activity level (reported in metabolic equivalents, MET, units) and clothing insulation (reported in clo levels) (van Hoof, 2008; Fanger, 1972).

It is well known that the PMV model has several limitations associated with generalisation to (1) populations other than healthy, young college students (i.e., those individuals tested by Fanger in his original climate chamber experiments that informed his comfort equation and PMV model); and (2) non‐steady‐state conditions (van Hoof, 2008). Despite these limitations, the PMV model provides a widely used empirical tool that can help identify the environmental conditions at which warm discomfort may onset, given specific assumptions. This approach can be particularly useful to identify the likely limits of perceptual heat tolerance as per IUPS criterion 1. We highlight this application in the example below, which we use for illustrative purposes. We wish to acknowledge that this example was developed using the CBE Thermal Comfort Tool, a free online tool for thermal comfort calculations and visualisations that implements thermal comfort calculations from international standards (Tartarini et al., 2020).

**FIGURE 2:**
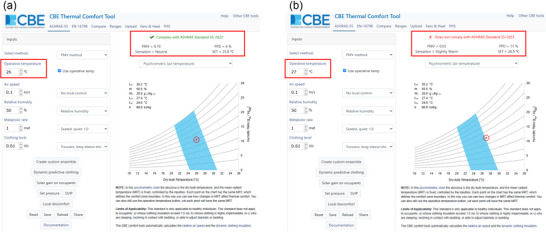
An example of the use of the PMV model to identify the environmental conditions at which thermal comfort is either maintained (a) or lost (b) given specific assumptions (see text for details on simulation parameters). This example was developed using the CBE Thermal Comfort Tool, a free online tool for thermal comfort calculations and visualisations that implements thermal comfort calculations from international standards (Tartarini et al., 2020).

In this example, we consider a healthy young adult, seated quietly in an indoor environment (i.e., activity level equivalent to 1 MET), wearing trousers and a long sleeve shirt (i.e., clothing insulation equivalent to 0.61 clo), exposed to a constant relative humidity of 50% and minimal airflow (i.e., air speed of 0.1 m s^−1^). Under this set of conditions, the PMV model identifies 26°C operative temperature (which is defined as the average of the mean radiant and ambient air temperatures, weighted by their respective heat transfer coefficients) as the maximum temperature beyond which comfort can no longer be maintained. Specifically, the PMV predicts that, given the conditions above, exposure to 26°C operative temperature will result in the population reporting feeling ‘neutral’ (which is compliant with the ASHRAE 55–2023 standard; Figure 2a); conversely, exposure to 27°C operative temperature would result in the population reporting feeling ‘slightly warm’ (which is non‐compliant with the ASHRAE 55–2023 standard; Figure 2b). With this example, one can infer that, within the set conditions above, the likely critical limit of perceptual heat tolerance for an individual aligning to the description above, coincides with an operative temperature of 26°C.

As noted earlier, the PMV model has several limitations, such that its outputs cannot be generalised to other populations, such as the elderly or clinical groups, who may experience age‐ and disease‐related changes in thermal sensation and comfort (Christogianni et al., 2023; Soebarto et al., 2019; van Hoof, 2008). Furthermore, the PMV model views occupants as passive recipients of thermal stimuli driven by the physics of the body's thermal balance with its immediate environment, and it does not consider how factors beyond fundamental physics and physiology, such as cultural, climatic, social and contextual dimensions of comfort, modify thermal sensation (de Dear, 2011; de Dear et al., 2020). These well‐established limitations prompted the development of the Adaptive Comfort model (de Dear & Brager, 1998) and its implementation within ASHRAE standard 55 (Olesen & Brager, 2004).

The fundamental premise of the adaptive model of comfort is that it views building occupants as ‘active systems’, who play a key role in creating their own thermal preferences by incorporating behavioural, physiological and psychological modes of thermal adaptation (de Dear & Brager, 1998). Operationally, a key advance of the adaptive model is that it quantifies the extent by which (thermal) adaption, due to exposure to prevailing outdoor ambient temperatures (which fluctuate seasonally and more evidently in naturally ventilated as opposed to mechanically conditioned buildings), increases the range of acceptable indoor temperatures beyond those predicted by the PMV model (de Dear & Brager, 1998). What this means in practice is that the model predicts higher limits of perceptual heat tolerance (as per IUPS criterion 1) than those identifiable by the PMV model. We highlight this comparison in the example below, which we use for illustrative purposes, leveraging the CBE Thermal Comfort Tool (Tartarini et al., 2020).

**FIGURE 3:**
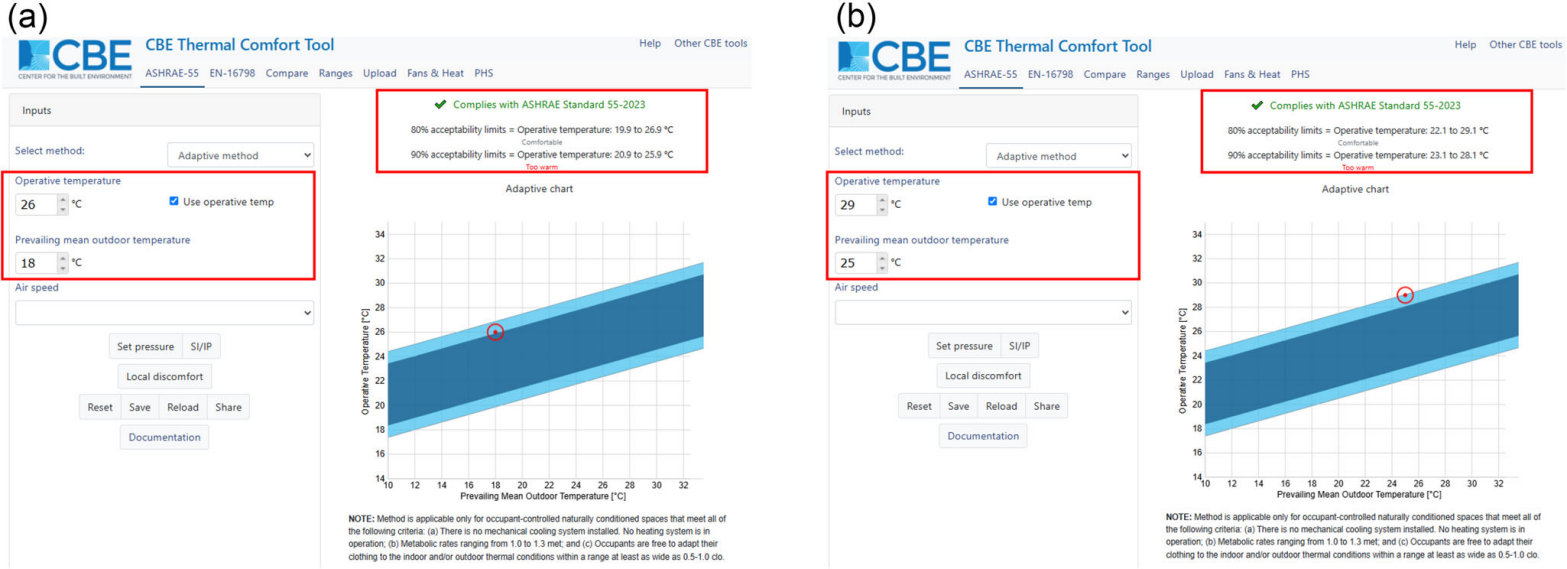
An example of the use of the Adaptive Comfort model to identify the environmental conditions at which thermal comfort is maintained in relation to lower (a) or higher (b) mean outdoor temperatures and given specific assumptions (see text for details on simulation parameters). This example was developed using the CBE Thermal Comfort Tool, a free online tool for thermal comfort calculations and visualisations that implements thermal comfort calculations from international standards (Tartarini et al., 2020).

In this example, we consider the same conditions as in the example above, that is, a healthy young adult, seated quietly in an indoor environment (i.e., activity level equivalent to 1 MET), wearing trousers and a long sleeve shirt (i.e., clothing insulation equivalent to 0.61 clo), exposed to a constant relative humidity of 50% and minimal airflow (i.e., air speed of 0.1 m s^−1^). According to the adaptive model, the range of operative temperatures within which comfort can be maintained will vary depending on the prevailing mean outdoor temperature. For example, if the prevailing mean outdoor temperature is 18°C (e.g., typical mean spring temperature in Sicily, Italy), then the range of indoor operative temperatures within which comfort can be maintained by the majority (80%) of the population is 19.9°C to 26.9°C (Figure 3a). As such, one can infer that, given this prevailing mean outdoor temperature, the likely critical limit of perceptual heat tolerance for an individual aligning to the description above, coincides with an operative temperature of 26°C. However, if the prevailing mean outdoor temperature is 25°C (e.g., typical mean summer temperature in Sicily, Italy), then the range of indoor operative temperatures within which comfort can be maintained by the majority (80%) of the population is 22.1–29.1°C (Figure 3b). As such, one can infer that, given this prevailing mean outdoor temperature, the likely critical limit of perceptual heat tolerance for an individual aligning to the description above, coincides with an operative temperature of 29°C.

The most important takeaway of the examples above is that the use of the Adaptive Comfort model highlights how the limits of perceptual heat tolerance are not fixed, but can be altered by the climatic context, past thermal experiences and current thermal expectations of building occupants. This consideration may not be entirely surprising to the thermal physiologist who is well accustomed to the perceptual effects of both heat acclimation and acclimatization, which include a reduction in thermal discomfort for a given ambient temperature (Park & Palmes, 1947; Périard et al., 2015; Sawka et al., 2011).

The complementary nature of these models (de Dear & Brager, 1998) indicates that the combined use of the PMV and Adaptive Comfort models can inform a reasonably reliable, empirically derived assessment of the critical environmental limits of perceptual heat tolerance in young, otherwise healthy adults. While those models can help with a reasonable prediction of ‘how hot is too hot’ perceptually for young adults under a given set of conditions, they are not designed to determine equivalent limits of physiological and functional heat tolerance, models of which are reviewed in the sections below.

## 4 EMPIRICAL MODELS OF PHYSIOLOGICAL HEAT TOLERANCE

The second IUPS criterion to define human heat tolerance is that associated with the maintenance of heat balance at particularly high ambient temperatures. As noted earlier, one could use this definition to identify ambient temperatures beyond which heat balance can no longer be maintained, and to describe them as operational limits to human physiological heat tolerance.

This approach has long been adopted in the study of the physiology of human body temperature regulation and of work limits in hot environments, with seminal research dating back to the 1960s and 1970s (Dukesdob & Henschel, 1973; Givoni & Goldman, 1972; Lind, 1963, 1963b, 1970; Lind et al., 1970; World Health Organization, 1969). To consider this dimension of human heat tolerance, one requires first a basic understanding of the autonomic physiology of human body temperature regulation and the biophysics of human heat balance. For recent reviews of these topics, the reader is referred to the excellent work of Cramer et al. (2022).

Briefly, autonomic responses underlying human body temperature regulation (i.e., changes in vasomotor and sudomotor tone and shivering and non‐shivering thermogenesis) are aimed at maintaining core body temperature within a narrow range, that is, 36–37°C, which is deemed optimal for cellular function (Cramer et al., 2022). The human body prevents core overheating by achieving heat balance, a dynamic thermal state which sees a balance between heat gains and heat losses from the body to the environment. Deep body (core) and skin (shell) temperatures are the principal variables driving the onset of the adaptive responses that regulate the balance between heat production and heat loss from the body to the environment (Kenny & Jay, 2013; Parsons, 2014). The conceptual heat balance equation summarises the biophysical and environmental factors involved in determining the heat exchanges between the body and the surrounding environment (i.e., thermal audit):

$$M-W=K+R+C+E_{\mathrm{sk}}\pm S$$

where *M* is the rate of metabolic energy production (W m^−2^), *W* is the rate of mechanical work (W m^−2^), *E* is the rate of evaporative heat loss (W m^−2^), *R* is the rate of radiative heat loss (W m^−2^), C is the rate of convective heat loss (W m^−2^), *K* is the rate of conductive heat loss (W m^−2^) and *S* is the rate of heat storage (W m^−2^).

The metabolic rate of the body (*M*) provides energy to perform mechanical work (*W*), and the net difference between the two (*M* − *W*) represents the amount of energy released by the body as heat. This value is always positive and represents the body heat production. To achieve thermal balance (i.e., *S* = 0), the heat produced by the body must be balanced by the heat released to the environment. This occurs via four main physical avenues: evaporation (*E*_sk_), radiation (*R*), convection (*C*) and conduction (*K*). Therefore, for heat balance (*S* = 0).

$$M-W-K-R-C-\;E_{\mathrm{sk}}=0$$

From a biophysical standpoint, if the value resulting from the above equation is positive, gains in body heat content occur, which will eventually lead to an increase in body temperature (Vanos et al., 2023). Physiologically, such changes in body heat content and temperature will trigger two main autonomic mechanisms of heat loss: cutaneous hyperaemia and the production of sweat, with the evaporation of sweat being the most powerful avenue for heat loss in humans (Cramer & Jay, 2016). At this point, it will be the combination of the thermal state of the body (i.e., resulting from an individual's rate of metabolic heat production and their maximum sweating capacity and skin wetness) *and* the environment's cooling power (i.e., the ability of the environment to absorb heat and moisture) that will determine whether the heat stress experienced is compensable or uncompensable (Cramer & Jay, 2016).

Compensable heat stress defines conditions where the rate of heat gain can be balanced by the rate of heat loss, meaning thermal balance can be achieved with an elevated (steady‐state) core temperature. For example, consider an individual who exercises in the heat and who has evaporative requirements for heat balance (*E*_req_) that (1) are below the environment's maximal capacity to absorb heat and moisture (*E*_max_) and (2) can be met by the individual's ability to achieve the required sweat rate and physical skin wetness (Cramer & Jay, 2016, 2019; Kenny & Jay, 2013). Conversely, uncompensable heat stress defines conditions where the current rate of heat production cannot be matched by the achievable rate of heat loss, whether due to limits in the body's capacity to dissipate heat or due to the environment's limited capacity to absorb heat (Cramer & Jay, 2016, 2019; Kenny & Jay, 2013). This condition is characterised by a continuous rate of heat storage, and consequently by a continuous rise in core temperature, which will ultimately result in heat stroke if unreversed (Cheung et al., 2000; Lind, 1963). For example, consider an individual who exercises in a warm and humid environment with evaporative requirements for heat balance (*E*_req_) that can be achieved by the individual's sweat rate, but which are greater than the environment's maximal capacity to absorb heat and moisture (*E*_max_). The fact that the compensability of heat stress is dependent on both the body's thermal state and the environment's cooling power allows modelling of the critical environmental limits beyond which heat balance can no longer be achieved for a given thermal state (i.e., rate of metabolic heat production and sweat rate). Hence, this approach can effectively identify environmental limits of physiological heat tolerance as per IUPS criterion 2 (Bernard et al., 2024; Epstein et al., 2017; Wyndham, 1974).

In this context, the recent work of Bernard et al. (2024) has provided the most comprehensive assessment of the critical environmental limits of physiological heat tolerance in young and older, otherwise healthy adults to date. The cornerstone of Bernard et al.'s approach is to derive the critical combination of air temperature and humidity at which an individual transitions from a state of compensable to uncompensable heat stress for a given metabolic rate. The authors’ approach is based upon data collected over several decades from their laboratories that have contributed to the development of psychometric charts identifying critical environment limits (Bernard et al., 2007, 2005; Kenney, 2020; Kenney & Zeman, 2002; Kenney et al., 1993, 1987; O'Connor, 1999). This model is therefore particularly useful to identify the likely limits of physiological heat tolerance as per IUPS criterion 2. We highlight this application in the examples below, which we use for illustrative purposes.

**FIGURE 4:**
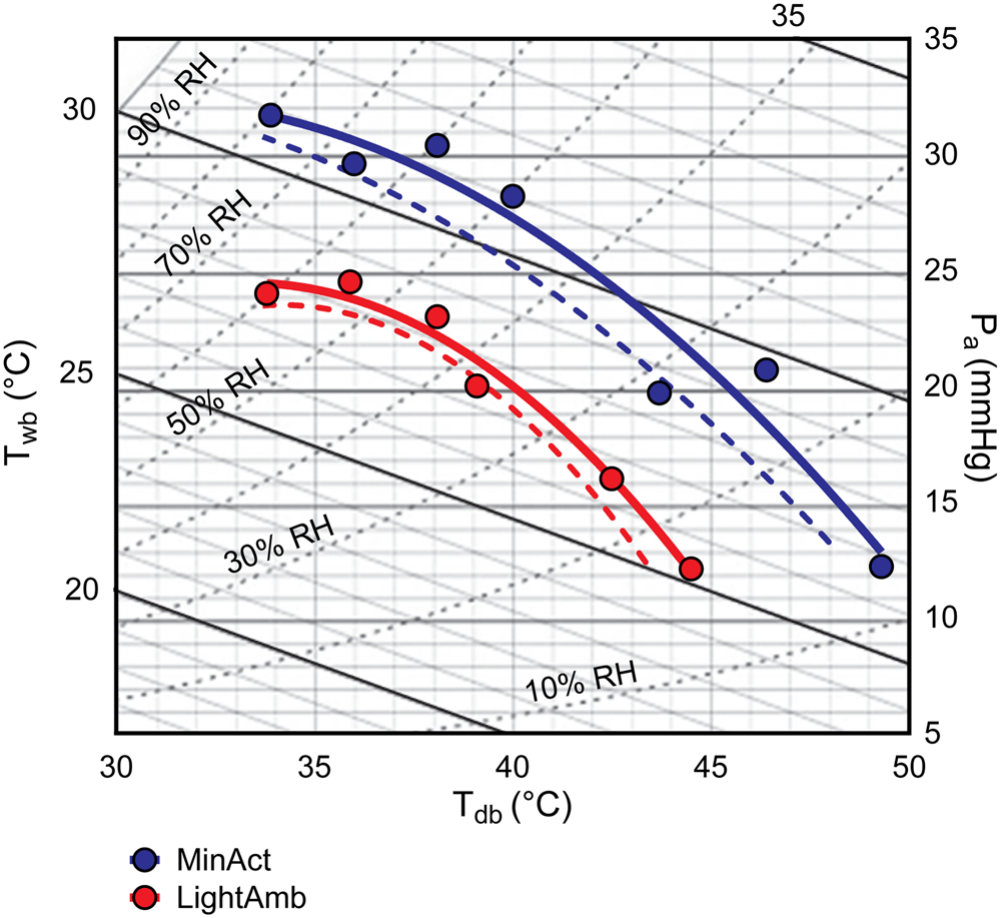
Psychrometric chart demonstrating critical environmental limits for young, healthy adults during light ambulation on a treadmill (lightamb; approx. 2.2 MET) and during minimal activity performed on a cycle ergometer (minact; approx. 1.3 MET). Smaller dashed lines denote the lower bounds of the 95% confidence interval for each condition. Reproduced with permission from Bernard et al. (2024).

In this example, we consider a healthy young adult, performing minimal daily‐living activities in an indoor (shaded) environment (i.e., activity level equivalent to 1.3 MET), wearing a cotton T‐shirt, shorts, socks and walking/running shoes (clo = ∼0.25) exposed to a constant relative humidity of 50% and minimal airflow (i.e., air speed of 0.1 m s^−1^). Under these conditions, the model by Bernard et al. (2024) identifies a dry bulb temperature (*T*_db_) of 39.5°C as the air temperature beyond which heat balance can no longer be maintained (Figure 4). With this example, one can infer that, within the set conditions above, the likely critical limit of physiological heat tolerance for an individual aligning to the description above coincides with an air temperature of 39.5°C.

Of note, the same group also developed empirical evidence on how the critical limits of physiological heat tolerance change in older individuals (≥65 years old) – identified in the work of Wolf et al. (2023). Ageing is well known to impair body temperature regulation, mostly via attenuations in sweat gland function and output, skin blood flow, cardiac output and an impaired ability to shunt blood from the viscera to the periphery (Kenney & Munce, 2003). For further information on the effect of ageing on the physiology of body temperature regulation, the reader is directed to two comprehensive reviews by Kenney and Munce (2003) and Kenny et al. (2010).

Using the same empirical approach as the one noted earlier, Wolf et al. (2023) found that ageing can shift the critical environmental limits for heat balance, thereby highlighting a change in physiological heat tolerance in older versus younger otherwise healthy adults.

**FIGURE 5:**
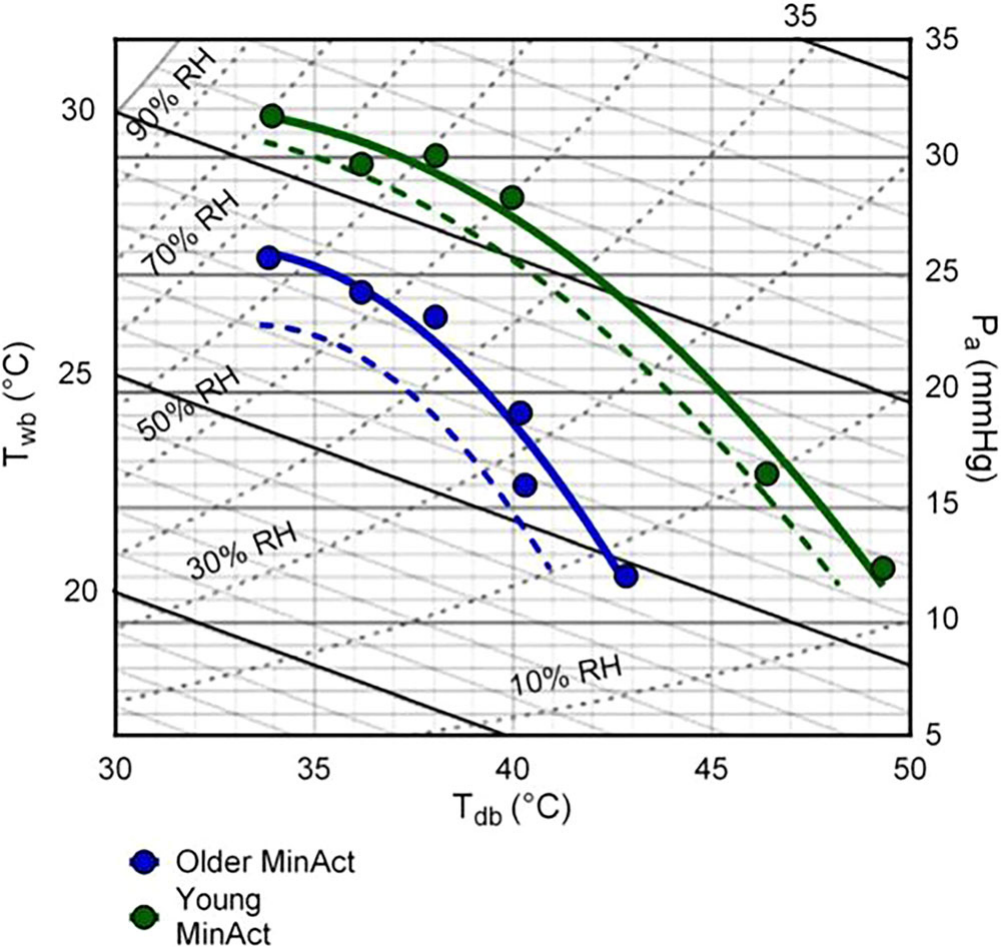
Psychrometric chart demonstrating critical environmental limits for young (23 ± 4 years) and older (71 ± 6 years) adults during minimal physical activity (minact). Critical environmental limits were significantly lower (i.e., shifted downwards and to the left; *P* < 0.0001) for older compared to young adults across all environmental conditions tested. Reproduced with permission from Wolf et al. (2023).

For example, using the same parameters as those of the example above (i.e., minimal daily‐living activities equivalent to ∼1.3 MET, wearing clo = ∼0.25, exposed to conditions with a relative humidity of 50% and air speed of 0.1 m s^−1^), the work by Wolf et al. (2023) identifies a dry bulb temperature of 36.7°C as the critical environmental limit for heat balance for a ≥65‐year‐old adult (Figure 5). Interestingly, this critical limit of physiological heat tolerance is ∼2.8°C lower than that identified for younger adults (Wolf et al., 2023), thereby clearly indicating a decline in physiological heat tolerance with ageing.

The models provided by Bernard et al. (2024) and Wolf et al. (2023) can inform a reasonably reliable, empirically derived assessment of the critical environmental limits of physiological heat tolerance in young and older (≥65 years old), otherwise healthy adults. While those models can help make a reasonable prediction of ‘how hot is too hot’ physiologically for young and older adults under a given set of conditions, they are not designed to determine equivalent limits of functional heat tolerance, models of which are reviewed in the sections below.

## 5 MODELS OF FUNCTIONAL HEAT TOLERANCE

The third IUPS criterion that defines human heat tolerance is that associated with the maintenance of ‘normal function’ or survival at body temperatures exceeding their normal range. As noted earlier, one could use this definition to identify body temperatures beyond which normal function or survival can no longer be maintained, and to describe them as operational limits to human functional heat tolerance. However, to evaluate this third dimension of human heat tolerance effectively, we believe that one must first define clear metrics that are representative of ‘normal function’, which in humans relate to the full operation of vital physiological processes associated with survival, as well as to normal tasks of daily living, including work.

As noted earlier, the challenge of defining ‘normal function’ effectively and consistently has not gone unnoticed by the IUPS Commission, which refers to the ambiguity associated with the use of the term ‘heat tolerance’ as synonymous with ‘heat endurance’, which indicates the ability to withstand a sustained stress (e.g., exercise in the heat) (Bligh & Johnson, 1973). Given that the IUPS definition references both the (relatively undefined) maintenance of ‘normal function’ and preservation of survival, we believe that ‘normal function’ could be broadly interpreted via metrics associated with *survivability*, *liveability* and *productivity*. These metrics cover the full spectrum of human function, including the ability to survive, live well and perform work in the heat. Endorsement of these metrics would also allow for operationalising an assessment of the limits of functional heat tolerance (IUPS criterion 3) by identifying those critical environmental conditions beyond which survival is not preserved, liveability is compromised and productivity begins to deteriorate. This approach would allow such operational limits of functional heat tolerance to be more directly comparable with those associated with perceptual and physiological heat tolerance (i.e., IUPS criteria 1 and 2).

If one takes the view that functional heat tolerance in humans relates to metrics of survivability, liveability and productivity, then two recent comprehensive models of (1) human survivability and liveability under heat stress (Vanos et al., 2023) and (2) heat stress‐induced changes in productivity (Foster et al., 2021) have provided critical environmental limits for functional heat tolerance in young and older, otherwise healthy adults.

In their model, Vanos et al. (2023) define the limit of survivability as reaching a core temperature of 43°C within a 3‐ to 6‐h exposure. Vanos et al.’s (2023) choice of this critical core temperature threshold (and related exposure duration) is motivated by (1) the need to identify a set of physiological conditions that would lead a human to experience heat stroke death from hyperthermia on 99.9% of occasions (i.e., the survivability metric) (Bouchama et al., 2022), and (2) the opportunity to compare critical environmental limit derived from this model with previous survivability models (Sherwood & Huber, 2010). The relevance of choosing a critical core temperature threshold to derive survivability limits is that it aligns clearly to the IUPS definition of functional heat tolerance (which considers the limits of survival at body temperatures exceeding their normal range, e.g., a core temperature >36–37°C) (Kenny & Jay, 2013).

An important advantage of the model by Vanos et al. (2023) is that it uses a more advanced approach to incorporate physiological principles of human thermal adaption to heat, which are rooted in the biophysics of whole‐body heat exchange and which were not fully captured by previous approaches (e.g., consider the 35°C wet bulb temperature model by Sherwood & Huber, 2010). Furthermore, the Vanos et al. (2023) model also illustrates the limits of liveability, defined as the [cit.] ‘maximum internal heat production, or level of physical activity, that a person can generate without a sustained rate of positive heat storage in the prevailing environment, thus allowing sustained work and play’. As a result, the model of Vanos et al. (2023) helps identify critical environmental limits for *liveability*. This is an important addition, as the concept of liveability (i.e., the ability to sustain a given level of work or play) also aligns with the IUPS definition of functional heat tolerance (which evaluates the limits of ‘normal function’, e.g., work and play, at body temperatures exceeding their normal range).

Vanos et al.’s (2023) approach to survivability and liveability can therefore be particularly useful to identify the likely limits of functional heat tolerance as per IUPS criterion 3. We highlight this application in the examples below, which we use for illustrative purposes.

**FIGURE 6:**
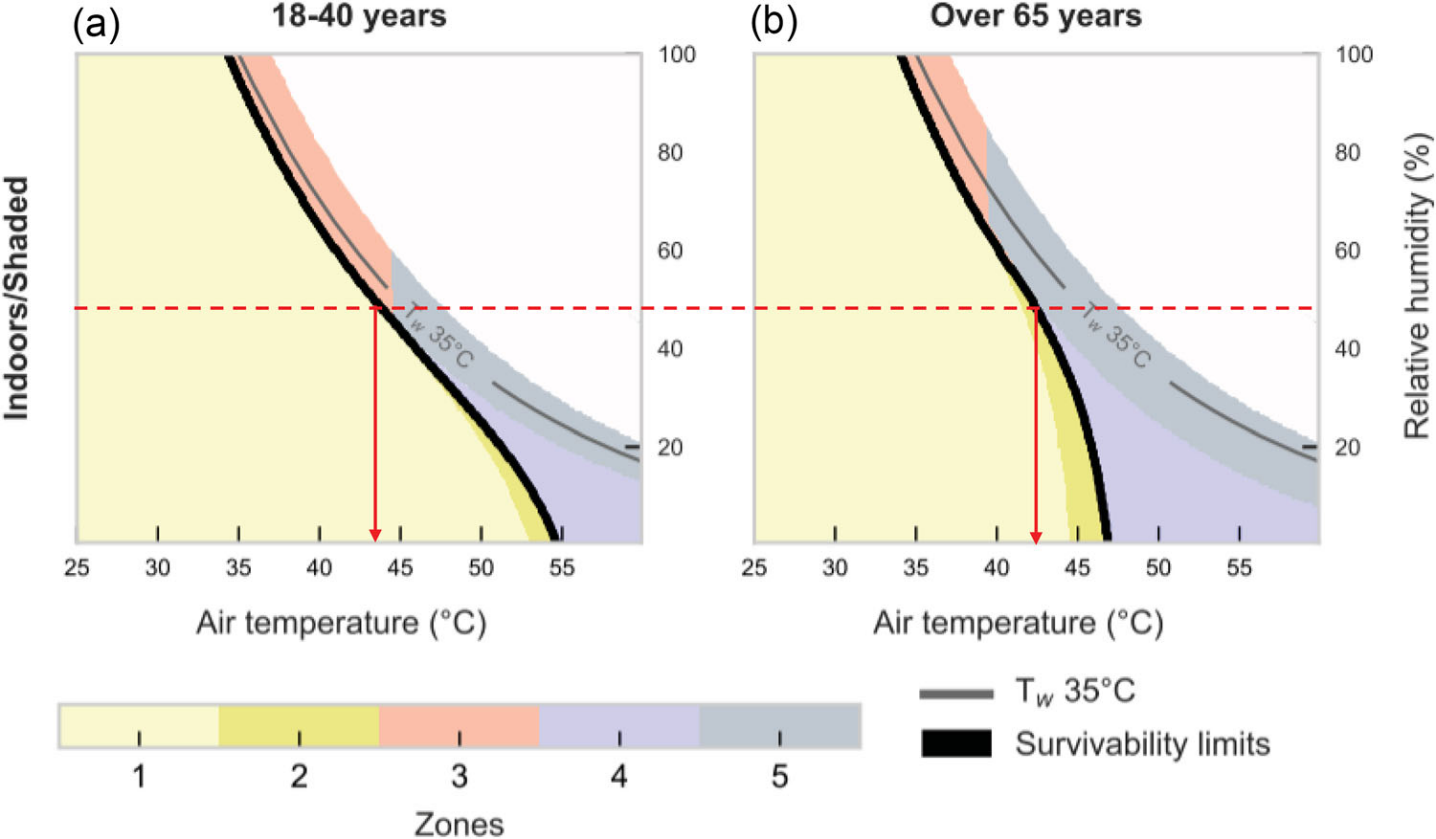
Psychrometric chart demonstrating critical environmental limits for survivability in young (a) and older (b) adults seated quietly in an indoor (shaded) environment (i.e., activity level equivalent to 1.5 MET), wearing no clothing. Reproduced with permission from Vanos et al. (2023).

In this example, we consider a healthy young adult, seated quietly in an indoor (shaded) environment (i.e., activity level equivalent to 1.5 MET), wearing no clothing (i.e., Vanos et al. assume clothing resistance being = 0 in their survivability assessment), exposed to a constant relative humidity of 50% and minimal airflow (i.e., air speed of 0.1 m s^−1^). Under this set of conditions, the model of Vanos et al. (2023) identifies 43.3°C air temperature as the maximum temperature beyond which survival cannot be maintained (i.e., beyond these conditions, a core temperature of 43°C will be reached in a 3‐ to 6‐h exposure, leading to heatstroke; Figure 6a).

Of note, the model of Vanos et al. (2023) also allows for evaluation of how the critical limits of survivability change in older individuals (≥65 years old) by incorporating assumptions of ageing‐induced declines in sweating capacity and associated impact on evaporative heat loss power. If one considers a change in age only in the example above, then the model of Vanos et al. (2023) identifies ∼42.5°C air temperature as the maximum temperature beyond which survival cannot be maintained by a ≥65‐year‐old individual (Figure 6b).

**FIGURE 7:**
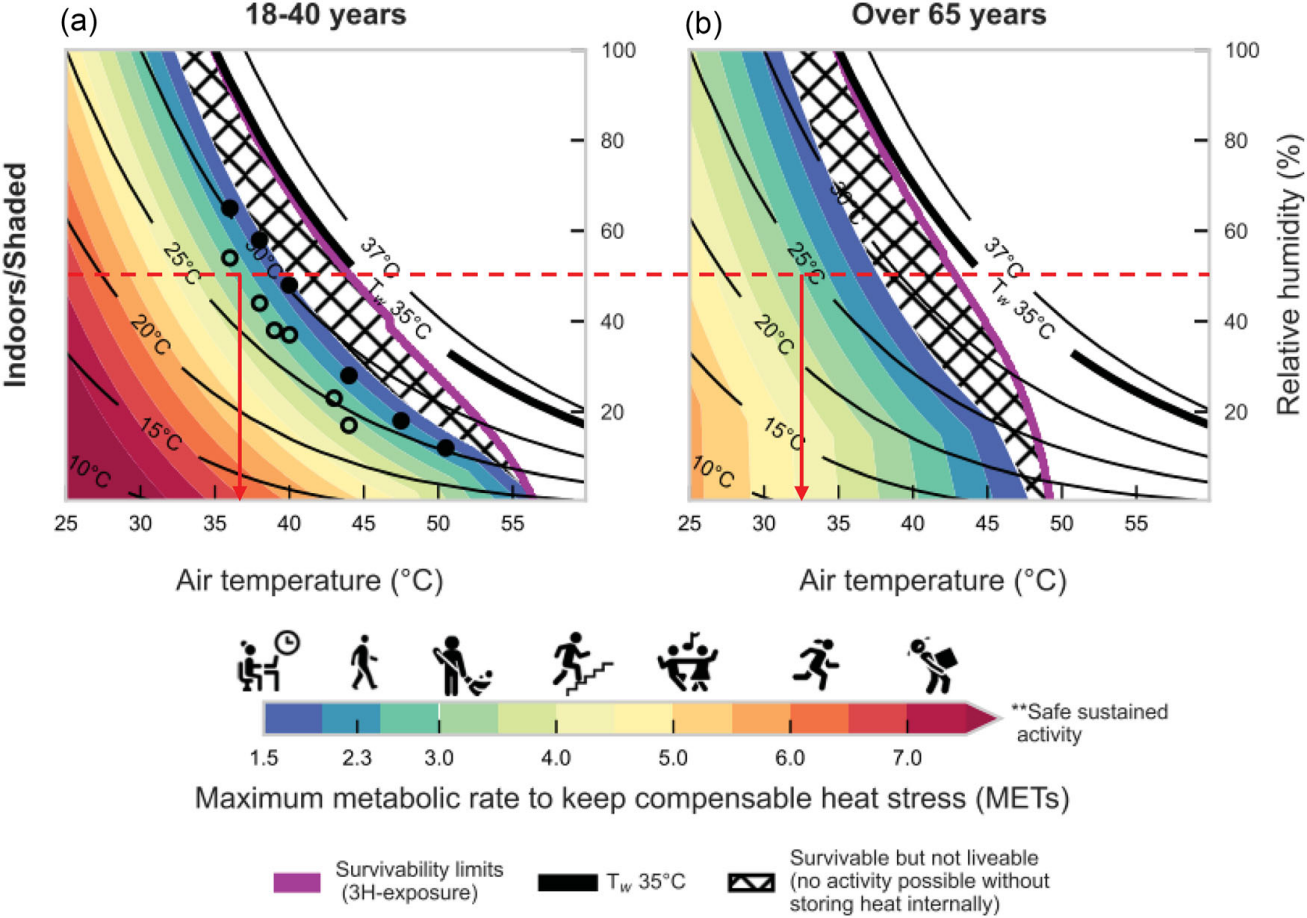
Psychrometric chart demonstrating critical environmental limits for liveability in young (a) and older (b) adults seated quietly performing light housework in an indoor (shaded) environment (i.e., activity level equivalent to 3 MET), while wearing light shorts and a cotton T‐shirt (i.e., equivalent to 0.36 clo). Reproduced with permission from Vanos et al. (2023).

The model of Vanos et al. (2023) expands the assessment of the critical limits of survivability to also accommodate the evaluations of shifts in critical thresholds associated with liveability, and we present a modified version of the example above to illustrate this point. For example, we consider the same healthy young adult as above, but this time performing light housework in an indoor (shaded) environment (i.e., activity level equivalent to 3 MET), while wearing light shorts and a cotton T‐shirt (i.e., equivalent to 0.36 clo), and being exposed to a constant relative humidity of 50% and minimal airflow (i.e., air speed of 0.1 m s^−1^). Under this set of conditions, the model of Vanos et al. (2023) identifies ∼36.5°C air temperature as the maximum temperature beyond which this activity level (∼3 MET) can no longer be sustained without rises in core temperature that will eventually lead to a core temperature of 43°C within 3–6 h (Figure 7a). The model of Vanos et al. (2023) also allows for evaluation of how the critical limits of liveability change in older individuals (≥65 years old) by incorporating assumptions of ageing‐induced declines in sweating capacity and associated impact on evaporative heat loss power. If one considers a change in age only in the example above, then the model of Vanos et al. (2023) identifies ∼32.5°C air temperature as the maximum temperature beyond which this activity level (∼3 MET) can no longer be sustained by a ≥65‐year‐old individual (Figure 7b).

The examples above indicate that the use of the model by Vanos et al. (2023) can inform a reasonably reliable assessment of the critical environmental limits of functional heat tolerance in younger and older, otherwise healthy adults, using metrics of survivability *and* liveability. While this model can help provide a reasonable prediction of ‘how hot is too hot for people’ functionally for young and older adults under a given set of conditions, it is not designed to assess productivity, which is intended as an additional and relevant metric of functional heat tolerance within conditions of liveability.

In this respect, the recent empirical model of Foster et al. (2021) has provided the most comprehensive laboratory‐based assessment of the impact of heat stress on physical work capacity (i.e., intended as a proxy of work output and labour productivity) across the widest range of thermal conditions tested to date.

In their work, Foster et al. (2021) define physical work capacity as ‘the maximum physical work output that can be reasonably expected from an individual performing moderate to heavy work over an entire shift’. Leveraging this definition, they developed a fixed cardiovascular strain protocol to model self‐pacing and associated work capacity loss under varying levels of heat stress, which resulted from exposures to a range of air temperatures (i.e., 25–50°C) and relative humidity (i.e., 20–80%). Specifically, they performed climatic chamber experiments where healthy young individuals walked on a treadmill that automatically adjusted its speed and incline to maintain a stable heart rate of 130 beats min^−1^ (identified as a meaningful indicator of moderate work strain); this was done on the basis that workers would pace themselves based on their heart rate/perceived exertion, resulting in similar values for working heart rate independent of the climate but at the cost of productivity (Foster et al., 2021).

**FIGURE 8:**
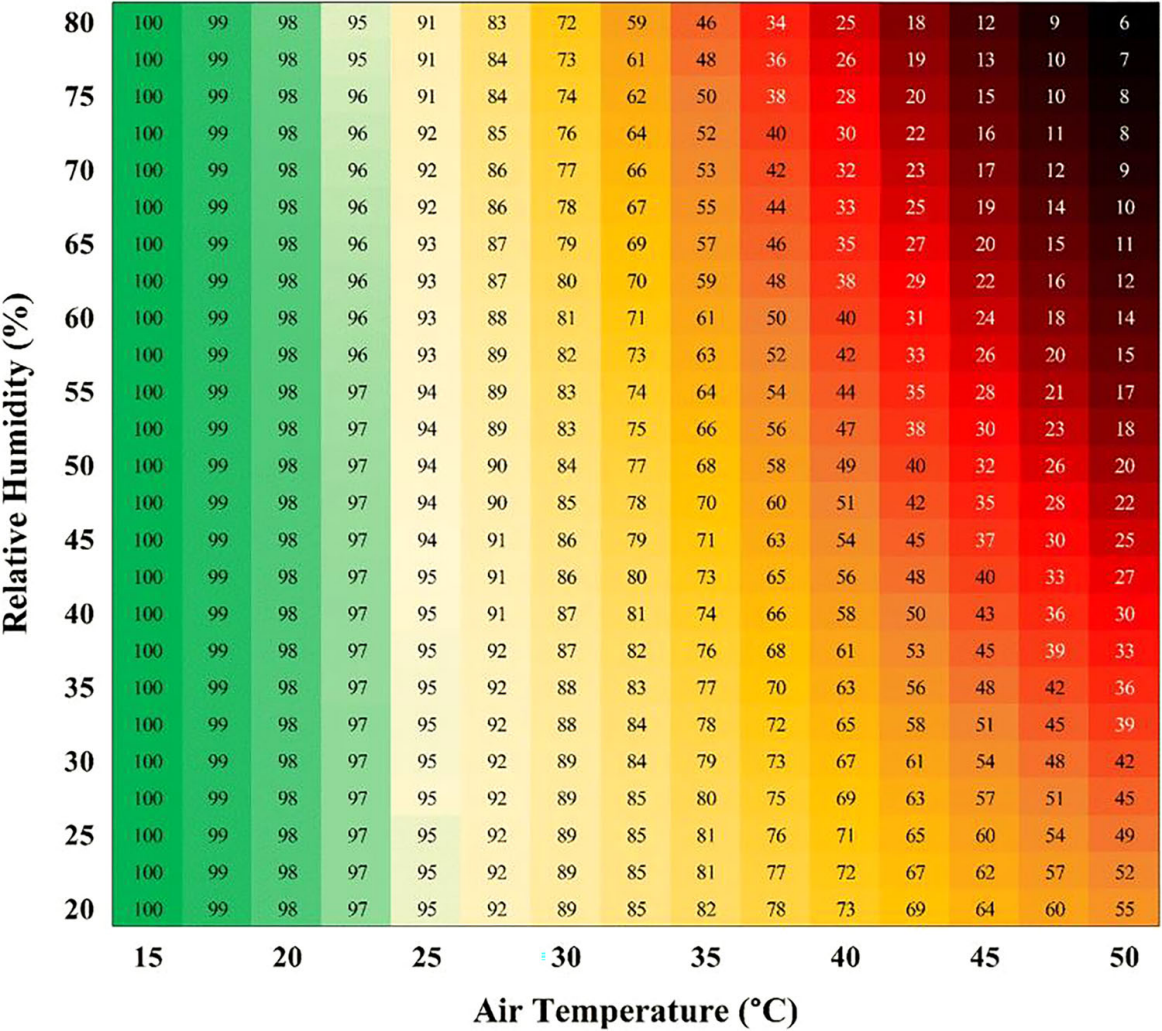
The change in physical work capacity percentage as a function of air temperature and relative humidity, as empirically derived by Foster et al. (2021). Values within the matrix indicate total physical work capacity as a percentage. Reproduced with permission from Foster et al. (2021).

This empirical work leveraged 338 work sessions in climatic chambers with low air movement, no solar radiation, spanning mild to extreme heat stress, to inform the development of predictive equations of physical work capacity based on air temperature and humidity. The model of Foster et al. (2021), therefore, allows for the assessment of the critical limits for work capacity loss (i.e., a proxy of functional heat tolerance as per IUPS criterion 3) (Figure 8). We highlight this application in the examples below, which we use for illustrative purposes.

In this example, we consider a healthy young adult, walking at a fixed heart rate 130 beats min^−1^, wearing a low clothing coverage ensemble (i.e., 0.26 clo), exposed to a constant relative humidity of 50% and minimal airflow (i.e., air speed of 0.1 m s^−1^). We also consider a 20% loss in physical work capacity as a meaningful effect size for the purpose of this example, as previously reported (Kenny et al., 2008). Under this set of conditions, the model of Foster et al. (2021) identifies ∼32.5°C as the air temperature resulting in such work capacity loss.

The example above indicates that the use of the model by Foster et al. (2021) can inform a reasonably reliable assessment of the critical environmental limits of functional heat tolerance in younger healthy adults, using metrics of physical work capacity associated with productivity.

At this point, it is important to note that the previously cited challenge of defining ‘normal function’ effectively and consistently in relation to the impact of heat stress does translate into a somewhat limited availability of models that truly capture the multi‐dimensional impact of heat stress on people's life and livelihoods across scales (e.g., temporal/cumulative impact) and dimensions (e.g., health inequalities, socio‐economic impact). The definition of ‘normal function’ in relation to heat stress is likely to require broader consideration to support a more holistic understanding of the impact of heat on people's function. This may also incorporate an evaluation of how insights derived from laboratory‐based experiments on critical limits may differ from ecologically valid field studies that consider freely behaving humans performing work under thermal stress (e.g., see recent work by Ioannou et al. (2025) on work‐capacity in thermally‐demanding occupational settings).

## 6 A HOLISTIC FRAMEWORK OF HUMAN HEAT TOLERANCE

The evidence reviewed in the preceding sections has highlighted the availability of several models (Bernard et al., 2024; de Dear & Brager, 1998; Foster et al., 2021; Fanger, 1972; Vanos et al., 2023) that can help provide a reasonable prediction of ‘how hot is too hot for people’ by identifying the critical air temperature for heat tolerance, given a specific set of environmental (e.g., relative humidity and air flow) and individual conditions (e.g., activity level and clothing insulation). When considered collectively, these models covered the full breadth of the IUPS criteria of heat tolerance, as they identified critical environmental limits across the perceptual, physiological and functional domains. However, each of the models highlighted addressed only a single domain of human heat tolerance. As far as the authors know, no model is available that is built on empirical evaluations of all domains of human heat tolerance concurrently and in the same participant cohort, likely due to the experimental challenges associated with such an approach.

The three IUPS criteria of heat tolerance are intrinsically connected, meaning that under real‐life scenarios of heat exposure, an individual is likely to concurrently experience changes in their comfort, heat balance and function. It follows that reliance on any of the models currently available alone (i.e., addressing either perceptual, physiological or functional tolerance) may only partially inform our understanding of the range of ecologically valid, critical environmental limits of heat tolerance that people are likely to experience under free‐living conditions. In other words, the complexity of the question ‘how hot is too hot for people’ lies in the fact that heat tolerance may vary largely based on whether the primary outcome of interest is perceptual, physiological or functional. Nevertheless, considering the similarities in participants’ characteristics (e.g., young adult cohorts) and activity/clothing insulation levels tested across the models reviewed here, we believe that the outcomes of these models can be reasonably integrated to evaluate the variation in human heat tolerance across perceptual, physiological and functional domains. Such an approach, albeit with limitations, could inform an initial holistic framework of human heat tolerance that more comprehensively characterises the full spectrum of possible responses to heat stress under free‐living conditions.

**FIGURE 9:**
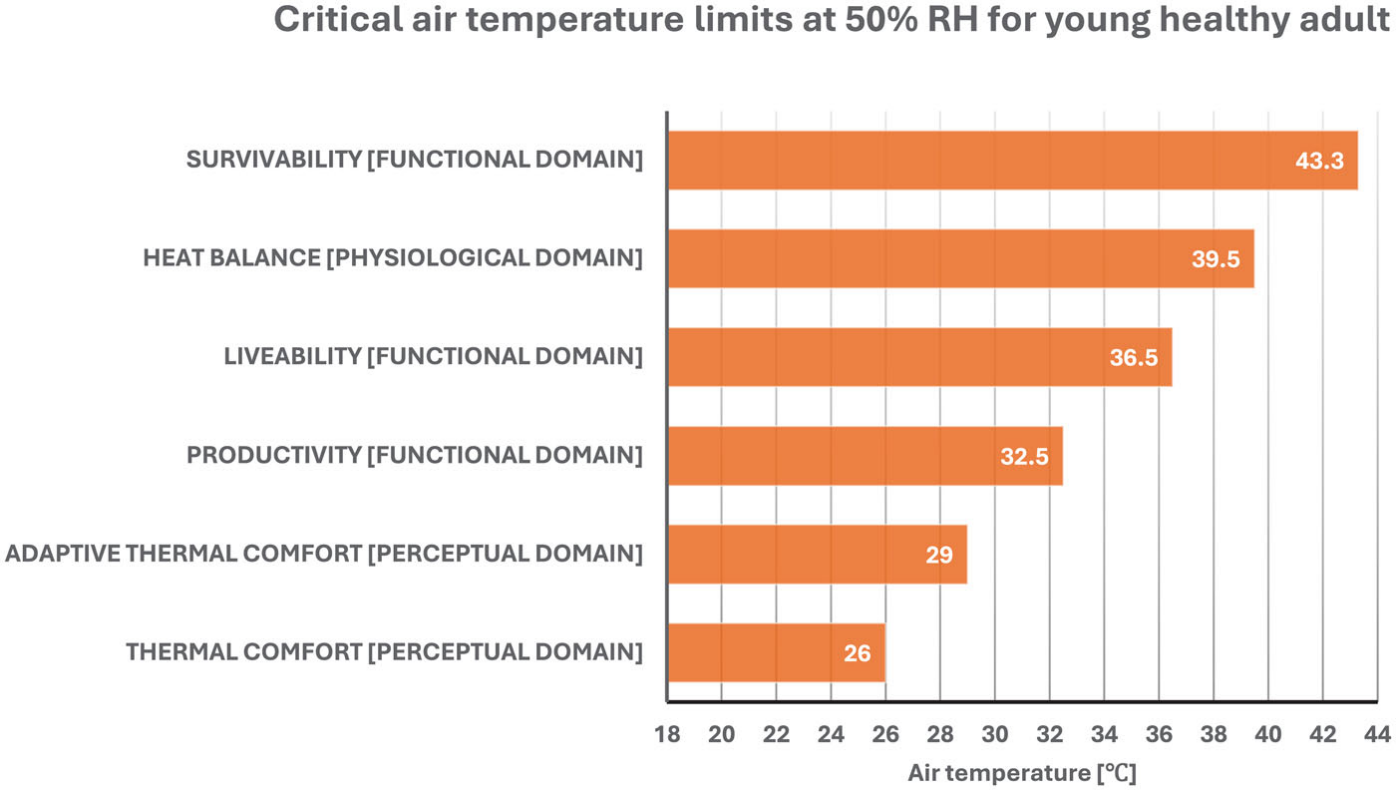
A visual representation of the critical air temperature limits of perceptual, physiological and functional heat tolerance leveraging current models for a healthy young adult, wearing no to light clothing (0–0.61 clo), and who is exposed to a constant relative humidity (RH) of 50% and minimal airflow.

Here we provide an example of this approach, which considers the given set of conditions presented in Sections 5, 6 and 5 of this review, and which aims to identify the critical air temperature beyond which perceptual, physiological and functional heat tolerance is surpassed for a healthy young adult, wearing no to light clothing (0–0.61 clo), and who is exposed to a constant relative humidity of 50% and minimal airflow (i.e., air speed of 0.1 m s^−1^) (note: for the liveability and productivity metrics, the models consider the same young adult doing light house working (∼3 MET) and walking at a fixed heart rate of 130 beats min^−1^). Figure 9 provides a visual representation of the critical air temperature limits of perceptual, physiological and functional heat tolerance for the example above.

Leveraging several models of heat tolerance across perceptual, physiological and functional domains, this example clearly indicates that the limits of human heat tolerance can vary between 26°C and 43°C, depending on the domain of interest. Overall, the critical air temperature limit for survival is the highest, whilst the limit for comfort is the lowest, with the limits associated with productivity and liveability being halfway between those. These observations above are well aligned to the conceptual architecture of the human thermoregulatory system and its underlying behavioural and autonomic heat‐defence mechanisms. For example, our example highlights that the critical limits of perceptual tolerance to warm discomfort occur at the lowest temperature within the analysed range (i.e., range: 26–29°C). This is not entirely surprising, given that thermal discomfort is a powerful trigger of behavioural responses to heat, which are well known to be engaged as a first line of defence from the heat, due to their greater capacity and energy efficiency than autonomic heat loss responses (e.g., sweating) (Schlader & Vargas, 2019). Our example also highlights that the limits of functional heat tolerance occur at higher air temperatures than those associated with perceptual tolerance, particularly if one considers productivity and liveability metrics (i.e., range: ∼32 to ∼36°C). This evidence indicates that a deterioration in, for example, physical work capacity will occur at higher air temperatures than those triggering the onset of warm discomfort, an observation which further underscores the importance of thermal sensation and behaviours as protective mechanisms to limit loss of function during heat exposures (Schlader et al., 2011). Finally, our example identified the highest critical limits for heat tolerance as those associated with physiological (heat balance) and survivability metrics (i.e. range: ∼39°C to ∼43°C). This evidence indicates that the limits of the human body's ability to maintain heat balance and avoid death by heat stroke will occur at higher air temperatures than those likely to trigger the onset of warm discomfort (i.e., perceptual domain) and a loss of physical work capacity (i.e., functional domain).

**FIGURE 10:**
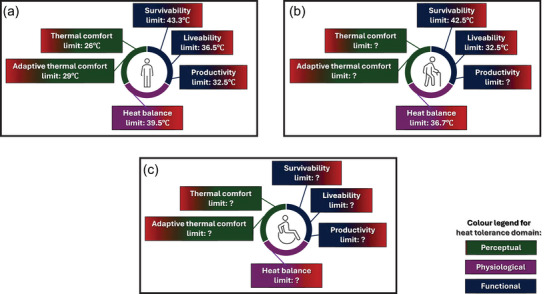
A conceptual, holistic framework of heat tolerance that considers perceptual, physiological and functional domains. (a) Critical environmental limits for air temperature for a healthy young adult, wearing no to light clothing (0–0.61 clo), and who is exposed to a constant relative humidity (RH) of 50% and minimal airflow. (b, c) The same environmental limits for the same scenario, yet in relation to a healthy older adult (b) and an individual with a chronic long‐term condition that may increase vulnerability to heat stress (e.g., an individual affected by multiple sclerosis) (c). Panels (b, c) highlight the limited availability of data that can help identify such critical environmental limits for individuals commonly acknowledged to be at greater risk of heat stress.

Taken together, the observations arising by combining models associated with all domains of heat tolerance, as evidenced in the example above, highlight a key take away. That is, human heat tolerance varies largely by domain of interest, and it is conceptually organised in line with the human body's ability to regulate body temperature via behavioural and autonomic responses. On this basis, we believe that to comprehensively address the question ‘how hot is too hot for people’ one must clearly define whether the primary domain of interest is to be perceptual, physiological and/or functional. Here we propose a conceptual, holistic framework of heat tolerance that could accommodate such nuanced considerations (Figure 10). This multi‐domain approach is particularly important if the applied goal of the question is informing policy and practice associated with thermal protection. Consider one of the examples provided in the introduction of this review, whereby an answer to the question ‘how hot is too hot’ may be sought by hospital managers to inform indoor air temperature limits in various areas of a hospital setting. One may consider how a specific indoor temperature limit may be adapted (i.e., being higher or lower) depending on whether the perceptual heat tolerance versus functional capacity of healthcare staff is to be prioritised during heatwave periods, if available infrastructure and/or personal cooling interventions are limited (Brooks et al., 2023). Our proposed holistic framework considers all domains of heat tolerance and it highlights the range of variation in relevant heat tolerance metrics, in order to broaden the range of adaptive opportunities that can be explored to increase the resilience of systems and people to heat stress (Jay et al., 2021).

It is important to note that the lack of comprehensive data on how each of the metrics assessed in each of the models presented in this review may vary with individual characteristics beyond those assessed (i.e., main data source arise from testing of healthy young adults) limits significantly the generalisability of the critical air temperatures highlighted in Figure 9 beyond healthy young adult cohorts; albeit we acknowledge that the Bernard et al. (2024) and the Vanos et al. (2023) models provide estimates of the shift in ‘physiological tolerance’ resulting from ageing (no such evidence is available for any perceptual domain nor other functional metrics, e.g., productivity). Nevertheless, we believe that our proposed framework could be used to interrogate such holistic questions associated with better understanding broader heat tolerance domains (i.e., including perceptual and functional) beyond physiological ones.

For example, Vanos et al. (2023) reported lower critical air temperature limits for survivability in older adults, owing to an assumption of compromised sweating capacity with older age; this was empirically demonstrated by Wolf et al. (2023), Bernard et al. (2024) and Guergova & Dufour (2011) who identified lower air temperature limits for physiological heat tolerance in older adults; however, it remains unclear how such critical limits relate to the perceptual domain, given the well‐known thermal sensitivity loss occurring with ageing (Figure 10b) (Guergova & Dufour, 2011). Similarly, the generalisability of the critical air temperatures highlighted in Figure 9 to vulnerable groups such as people with chronic health conditions (e.g., heat‐sensitive neurological disease) is also limited, given that no model has evaluated heat tolerance in clinical groups (Figure 10c). This highlights a gap in our understanding of how critical limits within the proposed framework may adapt in the presence of, for example, heightened perceptual sensitivity in heat‐sensitive neurological patients such as people with multiple sclerosis (Christogianni et al., 2023). Furthermore, it remains unclear how limits may also shift due to heat‐induced exacerbation of underlying cardiovascular and/or renal pathology in vulnerable groups (Barry et al., 2024).

Future studies may therefore consider leveraging our proposed holistic framework of heat tolerance (Figure 10) to empirically evaluate all domains of human heat tolerance concurrently, and in various participant cohorts. Ultimately, we believe that this approach will help comprehensively characterise the full spectrum of possible human responses to heat stress under free‐living conditions, thereby improving the effectiveness of person‐ and context‐centred heat‐protection strategies and interventions to increase heat resilience for all.

## 7 CONCLUSIONS

Human heat tolerance varies largely across perceptual, physiological and functional domains and is conceptually organised in line with the human body's ability to regulate body temperature via behavioural and autonomic responses. Our review of empirical models of heat tolerance indicates that to comprehensively address the question ‘how hot is too hot for people’, one must clearly define whether the domain of interest is to be perceptual, physiological and/or functional. Leveraging several models of heat tolerance across the three domains, we propose a holistic framework of human heat tolerance that more comprehensively considers the entirety of possible human responses to heat stress under free‐living conditions. We have highlighted the limitations of such an approach and call on researchers to evaluate empirically and concurrently all domains of human heat tolerance across a broad range of individuals, to fully identify how specific vulnerabilities may modify tolerance limits. We hope that this framework will inspire the design of new holistic research on human heat tolerance, which will ultimately inform heat resilience policies and interventions to protect human health and life quality under a warming climate.

## AUTHOR CONTRIBUTIONS

Davide Filingeri and Nuno Koch Esteves conceived and designed the approach to this review. Davide Filingeri and Nuno Koch Esteves drafted the manuscript and revised the manuscript for intellectual content. Both authors have read and approved the final version of this manuscript and agree to be accountable for all aspects of the work in ensuring that questions related to the accuracy or integrity of any part of the work are appropriately investigated and resolved. All persons designated as authors qualify for authorship, and all those who qualify for authorship are listed.

## CONFLICT OF INTEREST

The authors declare that they have no competing interests.

## A LITERATURE SEARCH STRING

### PUBMED (10 year filter)

#### ALL (563 hits)

“Humans”[MeSH] AND ((“Climate Change”[MeSH] OR “Extreme Heat”[MAJR] OR “Temperature”[MeSH] OR “Humidity”[MeSH] OR “Hot Temperature” OR “Body Temperature Regulation/physiology”[MeSH]) AND (tolerance[Title/Abstract] OR capacity[Title/Abstract] OR ability OR tolera*[Title/Abstract] OR regulat*[Title/Abstract] OR heat balance[Title/Abstract] OR thermoregulat*[Title/Abstract] OR survivability[Title/Abstract] OR liveability[Title/Abstract] OR (“Thermotolerance”[MAJR])) AND (model[Title/Abstract] OR models[Title/Abstract] OR definition[Title/Abstract] OR defining[Title/Abstract] OR theory[Title/Abstract] OR concept*[Title/Abstract] OR framework[Title/Abstract] OR representation[Title/Abstract] OR paradigm[Title/Abstract] OR assess*[Title/Abstract] OR approach[Title/Abstract])) AND ((physiol* OR health OR sweat OR core OR body OR heart rate OR skin OR cardiovascular OR survival OR liveability OR live OR surviv* OR critical OR limits OR critical environmental limit) OR (percept* OR thermal comfort OR comfort OR psychol* OR feel* OR sensation OR sensing OR perceiv* OR mental) (perceptual OR thermal comfort OR comfort OR psychol*) OR (thermal behaviour OR behaviour OR strateg* OR mitigate* OR fans OR fan OR fanning OR dous* OR mist OR spray OR air condition* OR cloth* OR water OR hydrat* OR dehydrat* OR shade OR cool OR seeking OR cool‐seeking OR cool seeking OR immersion OR wet)) NOT (cultiva* OR crop* OR plant* OR farm OR plastic*[Title/Abstract] OR vehicle[Title/Abstract] OR cancer OR pregnan* OR amphibian OR mouse[Title/Abstract] OR rat[Title/Abstract] OR soil OR erosion[Title/Abstract] OR food OR virus OR road OR antibiotic OR bacteri* OR pathogens OR vaping OR smoking OR catalyst OR toxi* OR dams OR river OR fish* OR reservoir OR deforestation OR drug OR dicarbon* OR sepsis OR nano* OR hypothermia[Title/Abstract] OR ion OR schizophrenia OR dementia OR Alzheimer OR pain OR Animal*).

### Physiology only (855 hits)

“Humans”[MeSH] AND ((“Climate Change”[MeSH] OR “Extreme Heat”[MAJR] OR “Temperature”[MeSH] OR “Humidity”[MeSH] OR “Hot Temperature” OR “Body Temperature Regulation/physiology”[MeSH]) AND (tolerance[Title/Abstract] OR capacity[Title/Abstract] OR ability OR tolera*[Title/Abstract] OR regulat*[Title/Abstract] OR heat balance[Title/Abstract] OR thermoregulat*[Title/Abstract] OR survivability[Title/Abstract] OR liveability[Title/Abstract] OR (“Thermotolerance”[MAJR])) AND (model[Title/Abstract] OR models[Title/Abstract] OR definition[Title/Abstract] OR defining[Title/Abstract] OR theory[Title/Abstract] OR concept*[Title/Abstract] OR framework[Title/Abstract] OR representation[Title/Abstract] OR paradigm[Title/Abstract] OR assess*[Title/Abstract] OR approach[Title/Abstract])) AND (physiol* OR health OR sweat OR core OR body OR heart rate OR skin OR cardiovascular OR survival OR liveability OR live OR surviv* OR critical OR limits OR critical environmental limit OR water OR hydrat* OR dehydrat*) NOT (cultiva* OR crop* OR plant* OR farm OR plastic*[Title/Abstract] OR vehicle[Title/Abstract] OR cancer OR pregnan* OR amphibian OR mouse[Title/Abstract] OR rat[Title/Abstract] OR soil OR erosion[Title/Abstract] OR food OR virus OR road OR antibiotic OR bacteri* OR pathogens OR vaping OR smoking OR catalyst OR toxi* OR dams OR river OR fish* OR reservoir OR deforestation OR drug OR dicarbon* OR sepsis OR nano* OR hypothermia[Title/Abstract] OR ion OR schizophrenia OR dementia OR Alzheimer OR pain OR Animal*).

### Perceptual only (302 hits)

“Humans”[MeSH] AND ((“Climate Change”[MeSH] OR “Extreme Heat”[MAJR] OR “Temperature”[MeSH] OR “Humidity”[MeSH] OR “Hot Temperature” OR “Body Temperature Regulation/physiology”[MeSH]) AND (tolerance[Title/Abstract] OR capacity[Title/Abstract] OR ability OR tolera*[Title/Abstract] OR regulat*[Title/Abstract] OR heat balance[Title/Abstract] OR thermoregulat*[Title/Abstract] OR survivability[Title/Abstract] OR liveability[Title/Abstract] OR (“Thermotolerance”[MAJR])) AND (model[Title/Abstract] OR models[Title/Abstract] OR definition[Title/Abstract] OR defining[Title/Abstract] OR theory[Title/Abstract] OR concept*[Title/Abstract] OR framework[Title/Abstract] OR representation[Title/Abstract] OR paradigm[Title/Abstract] OR assess*[Title/Abstract] OR approach[Title/Abstract])) AND (percept* OR thermal comfort OR comfort OR psychol* OR feel* OR sensation OR sensing OR perceiv* OR mental) NOT (cultiva* OR crop* OR plant* OR farm OR plastic*[Title/Abstract] OR vehicle[Title/Abstract] OR cancer OR pregnan* OR amphibian OR mouse[Title/Abstract] OR rat[Title/Abstract] OR soil OR erosion[Title/Abstract] OR food OR virus OR road OR antibiotic OR bacteri* OR pathogens OR vaping OR smoking OR catalyst OR toxi* OR dams OR river OR fish* OR reservoir OR deforestation OR drug OR dicarbon* OR sepsis OR nano* OR hypothermia[Title/Abstract] OR ion OR schizophrenia OR dementia OR Alzheimer OR pain OR Animal*).

### Behaviour only (497 hits)

“Humans”[MeSH] AND ((“Climate Change”[MeSH] OR “Extreme Heat”[MAJR] OR “Temperature”[MeSH] OR “Humidity”[MeSH] OR “Hot Temperature” OR “Body Temperature Regulation/physiology”[MeSH]) AND (tolerance[Title/Abstract] OR capacity[Title/Abstract] OR ability OR tolera*[Title/Abstract] OR regulat*[Title/Abstract] OR heat balance[Title/Abstract] OR thermoregulat*[Title/Abstract] OR survivability[Title/Abstract] OR liveability[Title/Abstract] OR (“Thermotolerance”[MAJR])) AND (model[Title/Abstract] OR models[Title/Abstract] OR definition[Title/Abstract] OR defining[Title/Abstract] OR theory[Title/Abstract] OR concept*[Title/Abstract] OR framework[Title/Abstract] OR representation[Title/Abstract] OR paradigm[Title/Abstract] OR assess*[Title/Abstract] OR approach[Title/Abstract])) AND (thermal behaviour OR behaviour OR strateg* OR mitigate* OR fans OR fan OR fanning OR dous* OR mist OR spray OR air condition* OR cloth* OR water OR hydrat* OR dehydrat* OR shade OR cool OR seeking OR cool‐seeking OR cool seeking OR immersion OR wet)) NOT (cultiva* OR crop* OR plant* OR farm OR plastic*[Title/Abstract] OR vehicle[Title/Abstract] OR cancer OR pregnan* OR amphibian OR mouse[Title/Abstract] OR rat[Title/Abstract] OR soil OR erosion[Title/Abstract] OR food OR virus OR road OR antibiotic OR bacteri* OR pathogens OR vaping OR smoking OR catalyst OR toxi* OR dams OR river OR fish* OR reservoir OR deforestation OR drug OR dicarbon* OR sepsis OR nano* OR hypothermia[Title/Abstract] OR ion OR schizophrenia OR dementia OR Alzheimer OR pain OR Animal*).

## SCOPUS (10 year filter)

### ALL (12,683 hits)

(TITLE‐ABS‐KEY (“Humans”) AND ((“Climate Change” OR “Extreme Heat” OR “Temperature” OR “Humidity” OR “Hot Temperature” OR “Body Temperature Regulation”) AND (tolerance OR capacity OR (ability W/1 tolera*) OR regulat* OR “heat balance” OR thermoregulat* OR survivability OR liveability OR “Thermotolerance”) AND (model OR models OR definition OR defining OR theory OR concept* OR framework OR representation OR paradigm OR assess* OR approach)) AND ((physiol* OR health OR sweat OR core OR body OR “heart rate” OR skin OR cardiovascular OR survival OR liveability OR live OR surviv* OR critical OR “limits” OR “critical environmental limit”) OR (percept* OR “thermal comfort” OR comfort OR psychol* OR feel* OR sensation OR sensing OR perceiv* OR mental) OR (“thermal behaviour” OR behaviour OR strateg* OR mitigate* OR fans OR fan OR fanning OR dous* OR mist OR spray OR “air condition*” OR cloth* OR water OR hydrat* OR dehydrat* OR shade OR cool OR “cool‐seeking” OR immersion OR wet)) AND NOT (cultiva* OR crop* OR plant* OR farm OR plastic* OR vehicle OR cancer OR pregnan* OR amphibian OR mouse OR rat OR soil OR erosion OR food OR virus OR road OR antibiotic OR bacteri* OR pathogens OR vaping OR smoking OR catalyst OR toxi* OR dams OR river OR fish* OR reservoir OR deforestation OR drug OR dicarbon* OR sepsis OR nano* OR hypothermia OR ion OR schizophrenia OR dementia OR alzheimer OR pain OR animal*) AND PUBYEAR > 2013 AND PUBYEAR < 2026) AND PUBYEAR > 2013 AND PUBYEAR < 2026.

### Physiological (11,371 hits)

(TITLE‐ABS‐KEY(“Humans”) AND ((“Climate Change” OR “Extreme Heat” OR “Temperature” OR “Humidity” OR “Hot Temperature” OR “Body Temperature Regulation”) AND (tolerance OR capacity OR (ability W/1 tolera*) OR regulat* OR “heat balance” OR thermoregulat* OR survivability OR liveability OR “Thermotolerance”) AND (model OR models OR definition OR defining OR theory OR concept* OR framework OR representation OR paradigm OR assess* OR approach)) AND (physiol* OR health OR sweat OR core OR body OR “heart rate” OR skin OR cardiovascular OR survival OR liveability OR live OR surviv* OR critical OR “limits” OR “critical environmental limit”) AND NOT (cultiva* OR crop* OR plant* OR farm OR plastic* OR vehicle OR cancer OR pregnan* OR amphibian OR mouse OR rat OR soil OR erosion OR food OR virus OR road OR antibiotic OR bacteri* OR pathogens OR vaping OR smoking OR catalyst OR toxi* OR dams OR river OR fish* OR reservoir OR deforestation OR drug OR dicarbon* OR sepsis OR nano* OR hypothermia OR ion OR schizophrenia OR dementia OR Alzheimer OR pain OR Animal*) AND PUBYEAR > 2013 AND PUBYEAR < 2026) AND PUBYEAR > 2013 AND PUBYEAR < 2026.

### Perceptual (7252 hits)

(TITLE‐ABS‐KEY(“Humans”) AND ((“Climate Change” OR “Extreme Heat” OR “Temperature” OR “Humidity” OR “Hot Temperature” OR “Body Temperature Regulation”) AND (tolerance OR capacity OR (ability W/1 tolera*) OR regulat* OR “heat balance” OR thermoregulat* OR survivability OR liveability OR “Thermotolerance”) AND (model OR models OR definition OR defining OR theory OR concept* OR framework OR representation OR paradigm OR assess* OR approach)) AND (percept* OR “thermal comfort” OR comfort OR psychol* OR feel* OR sensation OR sensing OR perceiv* OR mental) AND NOT (cultiva* OR crop* OR plant* OR farm OR plastic* OR vehicle OR cancer OR pregnan* OR amphibian OR mouse OR rat OR soil OR erosion OR food OR virus OR road OR antibiotic OR bacteri* OR pathogens OR vaping OR smoking OR catalyst OR toxi* OR dams OR river OR fish* OR reservoir OR deforestation OR drug OR dicarbon* OR sepsis OR nano* OR hypothermia OR ion OR schizophrenia OR dementia OR Alzheimer OR pain OR Animal*) AND PUBYEAR > 2013 AND PUBYEAR < 2026) AND PUBYEAR > 2013 AND PUBYEAR < 2026.

### Behavioural (9995 hits)

(TITLE‐ABS‐KEY(“Humans”) AND ((“Climate Change” OR “Extreme Heat” OR “Temperature” OR “Humidity” OR “Hot Temperature” OR “Body Temperature Regulation”) AND (tolerance OR capacity OR (ability W/1 tolera*) OR regulat* OR “heat balance” OR thermoregulat* OR survivability OR liveability OR “Thermotolerance”) AND (model OR models OR definition OR defining OR theory OR concept* OR framework OR representation OR paradigm OR assess* OR approach)) AND (“thermal behaviour” OR behaviour OR strateg* OR mitigate* OR fans OR fan OR fanning OR dous* OR mist OR spray OR “air condition*” OR cloth* OR water OR hydrat* OR dehydrat* OR shade OR cool OR “cool‐seeking” OR immersion OR wet) AND NOT (cultiva* OR crop* OR plant* OR farm OR plastic* OR vehicle OR cancer OR pregnan* OR amphibian OR mouse OR rat OR soil OR erosion OR food OR virus OR road OR antibiotic OR bacteri* OR pathogens OR vaping OR smoking OR catalyst OR toxi* OR dams OR river OR fish* OR reservoir OR deforestation OR drug OR dicarbon* OR sepsis OR nano* OR hypothermia OR ion OR schizophrenia OR dementia OR Alzheimer OR pain OR Animal*) AND PUBYEAR > 2013 AND PUBYEAR < 2026) AND PUBYEAR > 2013 AND PUBYEAR < 2026.
